# Risk of cervical cancer is not increased in Chinese carrying homozygous arginine at codon 72 of p53

**DOI:** 10.1038/sj.bjc.6690606

**Published:** 1999-08

**Authors:** H Y S Ngan, V W S Liu, S S Liu

**Affiliations:** Department of Obstetrics and Gynaecology, 6/Fl Professorial Block, University of Hong Kong, Pokfulam Road, Hong Kong

**Keywords:** p53 codon 72 polymorphism, cervical cancer, Chinese

## Abstract

Homozygous arginine at codon 72 (HA72) of p53 was found in 22% of normal cervices and 30.0% of cervical cancers and no significant difference was detected between normal and cervical cancer with or without HPV 16/18. There was no correlation between HA72 and risk of cervical cancer in Chinese. © 1999 Cancer Research Campaign


*British Journal of Cancer* (1999) **80**(11), 1828–1829
© 1999 Cancer Research Campaign
Article no. bjoc.1999.0606

Short Communication

### Risk of cervical cancer is not increased in Chinese carrying homozygous arginine at codon 72 of p53

**HYS Ngan, VWS Liu and SS Liu**

Department of Obstetrics and Gynaecology, 6/Fl Professorial Block, Queen Mary Hospital, Pokfulam Road, University of Hong Kong, Hong Kong

**Summary** Homozygous arginine at codon 72 (HA72) of p53 was found in 22% of normal cervices and 30.0% of cervical cancers and no significant difference was detected between normal and cervical cancer with or without HPV 16/18. There was no correlation between HA72 and risk of cervical cancer in Chinese.

**Keywords**: p53 codon 72 polymorphism; cervical cancer; Chinese

Cervical cancer is the commonest lower genital tract cancer found among Hong Kong Chinese. Human papillomavirus (HPV) infection has been found in over 80% of cervical cancers locally (Ngan et al, 1997). HPV plays an important role in cervical cancer due to degradation of p53 by the HPV E6 protein via the ubiquitine pathway (Werness et al, 1990). Storey et al (1998) found that women with homozygous arginine-72 (HA72) in p53 have a sevenfold increase in the risk of cervical cancer, perhaps because HA72 is more susceptible to E6-mediated degradation. However, subsequent studies in three Caucasian populations and one Japanese population have failed to support their findings (Hayes et al, 1998; Lanham et al, 1998; Minaguchi et al, 1998; Rosenthal et al, 1998). Since the frequency of p53 polymorphism varies amongst populations dwelling at different latitudes (Beckman et al, 1994), and Hong Kong is nearer the equator than the four reported studies, this study aims to determine the risk of cervical cancer in women with HA72 in p53 in Hong Kong Chinese.

### MATERIALS AND METHODS

### DNA samples

One hundred and two patients with cervical cancer and 68 women with normal cervices were studied. DNA samples (stored at –70°C) left from a previous study on p53 and HPV in cervical cancer (Ngan et al, 1997) were used in this study.

### PCR amplification of p53 codon 72 polymorphic alleles p53 arginine and proline sequences were separately amplified from each sample with primers as described by Storey et al (1998). Polymerase chain reaction (PCR) was done in a volume of 25 μl containing 100 ng total cellular DNA, 200 μM of each deoxynucleotide triphosphate, 0.625 U of Ampli *Taq* DNA polymerase (Perkins-Elmer Cetus), 1 × reaction buffer containing 1.25 mM magnesium ion. PCR was carried out in a Thermal Cycler (Perkin-Elmer Cetus) under conditions as follows: 40 cycles at 94°C for 30 s, 60°C for 30 s and 72°C for 30 s. The resulting PCR products were run in a 3% agarose gel and made visible under UV by ethidium bromide staining. The arginine PCR product was 141 bp and the proline PCR product was 177 bp.

### Detection of HPV

Results of HPV 16 and 18 from a previous study (Ngan et al, 1997) were used. The procedure used to detect HPV 16 and 18 E6 was essentially that described previously (Ngan et al, 1994). Briefly, DNA was extracted and subjected to two PCR assays using specific primers for HPV 16 and HPV 18 E6. PCR products were run on 4% agarose gels and blotted onto nylon membranes and hybridized with specific probes to HPV 16 and 18 E6 products. DNA from Caski and Hela were used as positive controls for HPV 16 and 18 respectively, and DNA derived from the C33 cell line and water were used as negative controls.

### Statistical analysis

Chi-square test was used to analyse nominal data. A $P$-value of $< 0.05$ was considered significant.

### RESULTS

HPV 16 or 18 E6 was detected in 78 samples (76.5%) of cervical cancer. Frequencies of arginine and proline p53 alleles in normal cervices and cervical cancer are shown in Table 1. In normal cervical tissue, 22% had HA72. This was not significantly different from cervical cancer with or without HPV infection having 32% and 25% of HA72 respectively ($\chi^2$ test, $P > 0.05$).

### DISCUSSION

The rate of arginine homozygosity in codon 72 polymorphism of p53 in normal cervical tissue of the local Chinese population was 0.22. This was much lower than that reported in other populations

*Received 5 January 1999*
*Revised 1 February 1999*
*Accepted 1 February 1999*

*Correspondence to:* HYS Ngan

**Table 1** Frequency of arginine (Arg) and proline (Pro) p53 alleles in normal cervices and in cervical cancer

| | No. | Pro | Arg | Pro/Arg |
|---|---|---|---|---|
| Cervical cancer | | | | |
| HPV-positive | 78 | 15 (0.19) | 25 (0.32) | 38 (0.49) |
| HPV-negative | 24 | 6 (0.25) | 6 (0.25) | 12 (0.50) |
| Normal cervices | 68 | 8 (0.12) | 15 (0.22) | 45 (0.66) |

in higher latitudes (Hayes et al, 1998; Lanham et al, 1998; Minaguchi et al, 1998; Rosenthal et al, 1998). In fact, Beckman et al (1994) found an increasing frequency of A2 allele (i.e. arginine) p53 polymorphism with increasing latitude and suggested that it may be ethnically related. Both Lanham et al (1998) and Rosenthal et al (1998) reported on UK population at a latitude of 52° where the HA72 rate was 0.57–0.68. Hayes et al (1998) reported on a population in Northern Holland living at a latitude of 52° where the HA72 rate was 0.57, a figure close to that in the UK. Minaguchi et al (1998) reported on a Japanese population living at a latitude of 36° where the HA72 rate was 0.36. Though both Chinese and Japanese are Orientals, the Japanese in that study were residing at higher latitude than the Hong Kong Chinese. In our study, Hong Kong is at a latitude of 23° and has 0.22, the lowest HA72 rate of any study. Combining the data from the above reports, our resulting observation is consistent with that of Beckman et al (1994). However, the biological significance of these findings is not apparent.

A recent study by Storey et al (1998) showed an increased susceptibility to degradation of p53 with HA72 by the HPV E6 protein and hence an increased risk of developing cervical cancer. In their study, a sevenfold increase in the risk of cervical cancer was found in HA72 carriers compared to heterozygous carriers. However, in our study as well as in other studies (Hayes et al, 1998; Lanham et al, 1998; Minaguchi et al, 1998; Rosenthal et al, 1998), no increase in the risk of cervical cancer in HA72 carriers was found. This may be the reason why cervical cancer is still quite common in our population despite the low HA72 rate. The clinical significance of HA72 in cervical cancer needs further exploration.

### REFERENCES

Beckman G, Birgander R, Sjalander A, Saha N, Holmberg PA, Kivela A and Beckman L (1994) Is p53 polymorphism maintained by natural selection? *Hum Hered* **44**: 266–270

Hayes VM, Hofstra RMW, Buys CHCM, Hollema H and van der Zee AGJ (1998) Homozygous arginine-72 in wild type p53 and risk of cervical cancer. *Lancet* **352**: 1756

Lanham S, Campbell I, Watt P and Gornall R (1998) p53 polymorphism and risk of cervical cancer. *Lancet* **352**: 1631

Minaguchi T, Kanamori Y, Matsushima M, Yoshikawa H, Taketani Y and Nakamura Y (1998) No evidence of correlation between polymorphism at codon 72 of p53 and risk of cervical cancer in Japanese patients with human papillomavirus 16/18 infection. *Cancer Res* **58**: 4585–4586

Ngan HYS, Stanley M, Liu SS and Ma HK (1994) HPV and p53 in cervical cancer. *Genitourin Med* **70**: 167–170

Ngan HY, Tsao SW, Liu SS and Stanley M (1997) Abnormal expression and mutation of p53 in cervical cancer – a study at protein, RNA and DNA levels. *Genitourin Med* **73**: 54–58

Rosenthal AN, Ryan A, Al-Jehani RM, Storey A, Harwood CA and Jacobs IJ (1998) p53 codon 72 polymorphism and risk of cervical cancer in UK. *Lancet* **352**: 871–872

Storey A, Thomas M, Kalita A, Harwood C, Gardiol D, Mantovani F, Breuer J, Leigh IM, Matlashewski G and Banks L (1998) Role of a p53 polymorphism in the development of human papillomavirus-associated cancer [see comments]. *Nature* **393**: 229–234

Werness BA, Levine AJ and Howley PM (1990) Association of human papillomavirus types 16 and 18 E6 proteins with p53. *Science* **248**: 76–79

*© 1999 Cancer Research Campaign*

